# cADPR Does Not Activate TRPM2

**DOI:** 10.3390/ijms23063163

**Published:** 2022-03-15

**Authors:** Winnie Maria Riekehr, Simon Sander, Jelena Pick, Henning Tidow, Andreas Bauche, Andreas H. Guse, Ralf Fliegert

**Affiliations:** 1 The Calcium Signalling Group, Department of Biochemistry and Molecular Cell Biology, University Medical Centre Hamburg-Eppendorf, 20246 Hamburg, Germany; w.riekehr@uke.de (W.M.R.); jelena.pick@stud.uke.uni-hamburg.de (J.P.); a.bauche@uke.de (A.B.); guse@uke.de (A.H.G.); 2 The Hamburg Advanced Research Center for Bioorganic Chemistry (HARBOR) & Department of Chemistry, Institute for Biochemistry and Molecular Biology, University of Hamburg, 22761 Hamburg, Germany; simon.sander@chemie.uni-hamburg.de (S.S.); henning.tidow@chemie.uni-hamburg.de (H.T.)

**Keywords:** transient receptor potential channel, cyclic adenosine 5′-diphosphate ribose, second messenger, calcium signalling

## Abstract

cADPR is a second messenger that releases Ca^2+^ from intracellular stores via the ryanodine receptor. Over more than 15 years, it has been controversially discussed whether cADPR also contributes to the activation of the nucleotide-gated cation channel TRPM2. While some groups have observed activation of TRPM2 by cADPR alone or in synergy with ADPR, sometimes only at 37 °C, others have argued that this is due to the contamination of cADPR by ADPR. The identification of a novel nucleotide-binding site in the N-terminus of TRPM2 that binds ADPR in a horseshoe-like conformation resembling cADPR as well as the cADPR antagonist 8-Br-cADPR, and another report that demonstrates activation of TRPM2 by binding of cADPR to the NUDT9H domain raised the question again and led us to revisit the topic. Here we show that (i) the N-terminal MHR1/2 domain and the C-terminal NUDT9H domain are required for activation of human TRPM2 by ADPR and 2′-deoxy-ADPR (2dADPR), (ii) that pure cADPR does not activate TRPM2 under a variety of conditions that have previously been shown to result in channel activation, (iii) the cADPR antagonist 8-Br-cADPR also inhibits activation of TRPM2 by ADPR, and (iv) cADPR does not bind to the MHR1/2 domain of TRPM2 while ADPR does.

## 1. Introduction

cADPR is a cyclic metabolite of NAD that was first found to release Ca^2+^ from intracellular stores in sea urchins by Hon Chong Lee in 1987 [1] but was later shown to act as Ca^2+^-releasing second messenger in a large variety of different cell systems (reviewed in [2,3]). Pharmacological data indicated that Ca^2+^ release occurs via activation of ryanodine receptors [4,5]. Besides releasing Ca^2+^ from intracellular stores, cADPR can also evoke Ca^2+^ influx (reviewed in [6]). In the human T-lymphoma cell line Jurkat, microinjection of cADPR leads to an increase in cytosolic Ca^2+^ that depends on extracellular Ca^2+^ and is not only blocked by the cADPR antagonist 8-NH_2_-cADPR but also by extracellular Zn^2+^ and the channel blocker SK&F-96365 [7]. In smooth muscle cells, cADPR and the nonhydrolysable cADPR agonist 3-deaza-cADPR stimulate Ca^2+^ entry [8]. This Ca^2+^ entry could either be a result of store depletion and activation of capacitative Ca^2+^ entry pathways or reflect activation of a Ca^2+^ permeable ion channel by cADPR.

In 2005, Kolisek et al. first reported that TRPM2, a nonselective, Ca^2+^-permeable member of the melastatin subfamily of TRP channels, can be activated by cADPR [9]. Before, TRPM2 activation was considered to be caused mainly by ADPR, a metabolite of NAD or cADPR. The cytosolic C-terminus of TRPM2 exhibits significant homology to NUDT9, an enzyme of the Nudix family that hydrolyses the pyrophosphate bridge of ADPR, which led to the identification of ADPR as the first TRPM2 agonist [10]. The cADPR concentration needed for TRPM2 activation was clearly supraphysiological (EC_50_ of 700 µM, while endogenous concentrations are in the low µM range [11]), but the authors of the study also demonstrated that cADPR could act synergistically with ADPR at much lower concentrations [2]. Interestingly, cADPR actions could be specifically blocked by 8-Br-cADPR (a cADPR antagonist), whereas AMP, one of the products of NUDT9, blocked ADPR-mediated activation of TRPM2 (IC_50_ of 70 µM), indicating that cADPR acts upon a binding site distinct from the NUDT9 homology domain (NUDT9H) [9]. Another study put this into question by showing that cADPR-mediated activation of TRPM2 does not only require body temperature (no current evoked by cADPR at 25 °C) but is also dependent on the NUDT9H domain [12]. In the following years, the question of cADPR-mediated activation of TRPM2 was nearly put to rest by two studies that showed that the effect of cADPR on TRPM2 is due to the contamination of commercial cADPR by ADPR, and that the activating effect of cADPR vanishes when contaminating ADPR is hydrolysed (without affecting cADPR) by nucleotide pyrophosphatase [13,14] (see also our recent, more extensive review on the topic [15]).

Recent developments again raised the question of whether cADPR could be a direct TRPM2 modulator. Seminal work from Kühn and coworkers on the TRPM2 orthologue of *Nematostella vectensis* (nvTRPM2) showed that the NUDT9H domain in this channel is not required for activation by ADPR and instead hydrolyses the agonist, which was a first indication that the initial assumption that the NUDT9H domain is solely responsible for gating of TRPM2 is incorrect [16]. The first cryo-EM structure of TRPM2 from zebrafish (drTRPM2) then revealed a so-far unrecognised nucleotide-binding site in the MHR1/2 domain of the channel [17]. Interestingly, the ADPR molecule in the MHR1/2 domain assumes a horseshoe-like conformation that resembles the cyclic structure of cADPR (PDB: 6DRJ). This horseshoe-like conformation was confirmed in a later cryo-EM structure of human TRPM2 (PDB: 6PUS, [18]). The ADPR found in the NUDT9H domain of human TRPM2, in contrast, assumes an elongated conformation. The structure of human TRPM2 was also determined in the complex with the cADPR antagonist 8-Br-cADPR (PDB: 6PUU). Matching the other observations, 8-Br-cADPR was found in the MHR1/2 domain but not in the NUDT9H domain of the channel. While this could point towards a role of the MHR1/2 domain as a cADPR-binding site, another recent study showed effects of purified cADPR on TRPM2 and proposed that cADPR binds to the NUDT9H domain based on molecular dynamics simulations, mutagenesis studies, and surface plasmon resonance measurements (SPR) [19].

These developments led us to revisit the role of cADPR in TRPM2 activation. We tested cADPR, which we checked for purity by HPLC, for potential activation of TRPM2 under a variety of conditions, introduced mutations into the MHR1/2 and NUDT9H domain to analyse their role in agonist activation, and determined the binding of the MHR1/2 domain of TRPM2 to ADPR and cADPR.

## 2. Results

### 2.1. cADPR Does Not Activate Human TRPM2

**Figure 1:**
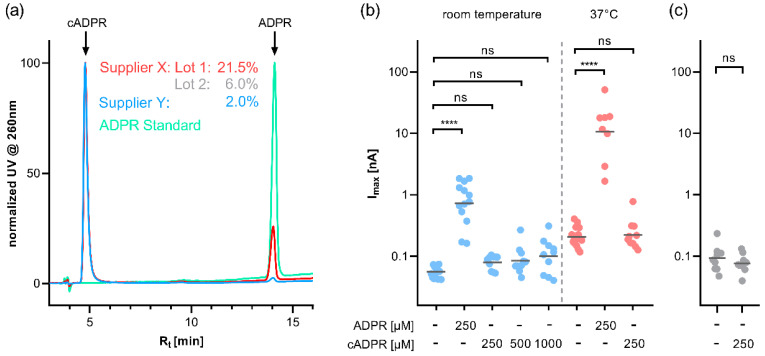
(**a**) HPLC analysis of cADPR from different suppliers (red, grey and blue). Due to the varying amount of cADPR in the preparations, the chromatograms have been normalised to the cADPR peak to illustrate the different fractions of ADPR in the preparations. The ADPR standard (green) is shown for comparison. (**b**) cADPR does not activate human TRPM2 at either room temperature or at 37 °C. HEK293 cells with stable expression of human TRPM2 were infused via the patch pipette with an intracellular solution buffered to a Ca^2+^ of 200 nM with EGTA and containing either 250 µM ADPR or 250 µM cADPR. Cells were kept at either room temperature (blue) or 37 °C (red) by continuous perfusion with bath solution. The bath solution contained NMDG instead of Na^+^. (See Supplementary Material Figure S1 for representative time courses and a schematic representation of the voltage ramp) (**c**) cADPR does not activate human TRPM2 in a bath solution with NaCl. At room temperature, TRPM2-expressing HEK293 cells were infused with an intracellular solution that contained either no nucleotide or 250 µM cADPR. Max currents at +15 mV from repetitive voltage ramps are shown on a log scale. Log transformed data were tested against the respective control by one-way ANOVA followed by post hoc *t* test with Bonferroni correction (**b**) or two-tailed Student’s *t* test (**c**), the mean of the log-transformed data is indicated by a horizontal bar (**** *p* < 0.0001, ns not significant).

Since cADPR is prone to both enzymatic and nonenzymatic hydrolysis, we analysed three different lots of cADPR obtained from two different vendors using ion-pair RP-HPLC [5,6]. These preparations did not only differ significantly in cADPR content but especially in the fraction of ADPR (2.0 to 21.5%, Figure 1a). For all further experiments regarding TRPM2 activation, we used the cADPR preparation with the highest cADPR and lowest ADPR content. The cADPR concentration was confirmed by completely hydrolysing cADPR to ADPR by heating it to 95 °C for 1 h and quantifying the resulting ADPR by HPLC against a standard that has been prepared by weighing in solid ADPR sodium salt.

To test cADPR for its activity towards TRPM2, we used a HEK293 cell line with stable expression of human TRPM2 we generated previously [20]. To avoid rupture of the cells due to excessive inward current, the bath solution contained N-methyl-d-glucamine (NMDG) instead of NaCl. Activation of TRPM2 was facilitated by clamping the Ca^2+^ in the pipette solution to 200 nM using EGTA.

cADPR has been described to activate TRPM2 at concentrations above 100 µM with an EC_50_ of 700 µM at room temperature [2]. We thus applied cADPR at 250 µM, 500 µM, and 1 mM via the patch pipette. Under our testing conditions, the application of cADPR did not significantly increase the maximum whole-cell current compared to the buffer control (57 pA ± 3 pA, *n* = 13). The application of 250 µM ADPR in the pipette, in contrast, resulted in significantly higher outward currents (940 pA ± 164 pA, *n* = 13) compared with the buffer control (Figure 1b left).

In contrast to Kolisek and coworkers, Togashi et al. observed activation of human TRPM2 by cADPR only at body temperature (37 °C) but not at 25 °C [7]. To investigate the effect of cADPR at 37 °C, we made sure to adjust our buffers to the increased temperature, since the chemical equilibria of both the pH buffer and the Ca^2+^/EGTA buffer are affected by temperature, and TRPM2 is highly sensitive to changes in both pH and Ca^2+^ [8,9,10]. During the whole-cell patch experiments, we observed an increase in the maximum outward current in the buffer control from 57 pA at room temperature to 219 pA ± 20 pA (*n* = 16) at 37 °C. Application of 250 µM cADPR did not significantly further increase this current (262 pA ± 60 pA, *n* = 10), whereas 250 µM ADPR resulted in a significantly higher current (16.5 nA ± 5.5 nA) compared with the buffer control at 37 °C. Of note, a roughly 18-fold higher current as compared with room temperature control was observed at 250 µM ADPR (Figure 1b, right half of the panel), comparable to what we observed previously in HEK293 cells transiently transfected with an expression vector for human TRPM2 [11].

To exclude that the lack of effect of cADPR was due to the absence of Na^+^ in the bath solution, we replaced the extracellular buffer solution with a buffer solution containing NaCl instead of NMDG. While under these conditions, most of the cells infused with 250 µM ADPR in the pipette solution ruptured during the recording, probably as a result of the rush of inward current, and the infusion of 250 µM cADPR did not result in a significant increase in maximum whole-cell current compared to the buffer control (40 pA ± 8 pA vs. 48 pA ± 4 pA, both *n* = 10) (Figure 1c).

**Figure 2:**
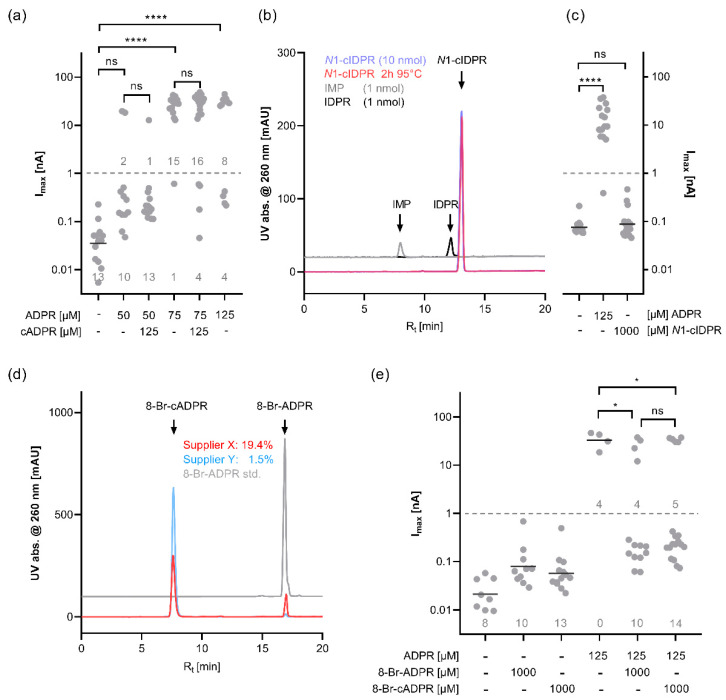
(**a**) cADPR does not enhance the TRPM2 current at threshold concentrations of ADPR. HEK293 cells with stable expression of human TRPM2 were infused via the patch pipette with an intracellular solution with weak Ca^2+^ buffering (100 µM EGTA) and containing varying concentrations of ADPR with or without 125 µM cADPR. During the recording, cells were kept at room temperature. With ADPR in the pipette solution, the currents show a bimodal distribution, with some cells showing a slight increase in current compared with the control while others exhibit a current two orders of magnitude higher. Log-transformed data were tested using Kruskal–Wallis test with Dunn’s correction (**** adj. *p* < 0.0001, ns = not significant) (**b**) HPLC analysis of *N*1-cIDPR (blue), *N*1-cIDPR that has been incubated at 95 °C for 2 h (red), and standards of potential breakdown products IMP (grey) and IDPR (black). (**c**) The nonhydrolysable cADPR analogue *N*1-cIDPR does not activate human TRPM2. TRPM2-expressing HEK293 cells were infused with a pipette solution containing either no nucleotide or either ADPR or *N*1-cIDPR. Log-transformed data were tested using Kruskal–Wallis test with Dunn’s correction (**** adj. *p* <0.0001, ns = not significant) (**d**) HPLC analysis of 8-Br-cADPR from two different suppliers (red and blue) and an 8-Br-ADPR standard (grey). The numbers indicate the fractional peak area of the 8-Br-ADPR peak in the analysed 8-Br-cADPR. (**e**) 8-Br-ADPR and 8-Br-cADPR reduce the ADPR-induced current in TRPM2-expressing HEK293 cells. Cells were infused with a pipette solution that contained no nucleotide, 125 µM ADPR, 1 mM 8-Br-ADPR, 1 mM 8-Br-cADPR, or a combination thereof. For some conditions, the horizontal bar indicates the mean of the log-transformed currents. Due to the bimodal distribution, the fraction of cells responding to a current above and below 1 nA were compared using Fisher’s exact test (* *p* < 0.05, ns = not significant).

In a recent study, Yu et al. described cADPR evoked currents in TRPM2-expressing HEK293 cells using a pipette solution with little Ca^2+^ buffering [12]. We thus also reduced the Ca^2+^ buffering in our system and used a nominally Ca^2+^ free buffer solution with 100 µM EGTA. Under these conditions, the channel behaved somewhat differently; instead of the continuous distribution of current amplitudes we observed in the previous experiments, we noticed a bimodal distribution of current amplitudes when we added nucleotides to the pipette solution. Some cells showed a current slightly higher than the buffer control, whereas other cells exhibited a current that was roughly 100-fold higher. We attributed this behaviour to positive feedback by Ca^2+^ ions entering through the open channel and sensitizing the channel to ADPR [21,22,23]. This feedback loop is largely repressed under conditions of high Ca^2+^ buffering but leads to an “all or nothing” response when the Ca^2+^ buffering is low. The threshold for TRPM2 activation by ADPR was apparently between 50 µM and 75 µM, at 50 µM, only 2 out of 10 cells showed a current larger than 1 nA, whereas, at 75 µM, 15 out of 16 cells showed a current of this amplitude. In contrast to what would be expected if cADPR acts synergistically with ADPR [9], addition of 125 µM cADPR did not significantly alter the current induced by ADPR alone, neither at the subthreshold concentration (w/o cADPR: 3.3 nA ± 2.1 nA, *n* = 12 vs. w cADPR: 1.1 nA ± 0.9 nA, *n* = 14) nor at the higher concentration (*w*/*o* cADPR: 26.7 nA ± 2.7 nA, n = 20 vs. w cADPR: 25.7 nA ± 3.6 nA, *n* = 16) (Figure 2a). In addition, we tested *N*1-cIDPR, a cADPR analogue that has been identified as cADPR agonist with almost identical EC_50_ as compared to cADPR [24,25]. Further, *N*1-cIDPR was shown to be resistant to hydrolysis (Figure 2b, no hydrolysis even after heating to 95 °C for 2 h) [26]. Of note, the addition of 1 mM *N*1-cIDPR to the pipette solution did not result in a higher current than observed with the buffer solution alone (81 pA ± 10 pA, *n* = 14 vs. 111 pA ± 23 pA, *n* = 19), whereas the addition of ADPR to the pipette solution significantly increased the current (Figure 2c).

### 2.2. 8-Br-ADPR and 8-Br-cADPR Both Inhibit Activation of Human TRPM2 by ADPR

In the absence of any observable effect of cADPR on human TRPM2, we were interested in whether 8-Br-cADPR, which had been shown to selectively inhibit activation of the channel by cADPR [9] and to bind to the N-terminal MHR1/2 domain [18], would have any effect on TRPM2 activation. Since 8-Br-cADPR, similar to cADPR, is prone to both enzymatic and nonenzymatic hydrolysis [27,28], and 8-Br-ADPR is a TRPM2 antagonist in its own regard [20,29], we again tested commercial 8-Br-cADPR preparations from two different vendors. The preparations differed both in content and purity. The purer preparation contained 1.5% 8-Br-ADPR (Figure 2d). We confirmed the concentration of 8-Br-cADPR in this preparation by completely hydrolysing 8-Br-cADPR to 8-Br-ADPR by heating to 95 °C for 2 h. The concentration of 8-Br-ADPR was then determined by ion-pair RP-HPLC using an 8-Br-ADPR standard, allowing us to establish the concentration of the 8-Br-cADPR solution before use.

When 1 mM of either 8-Br-ADPR or 8-Br-cADPR was applied via the patch pipette, they resulted in a slight increase in current compared with the buffer alone (Figure 2e). For 8-Br-ADPR, this was not unexpected as it was shown to act as a low-affinity partial agonist of TRPM2 [30]. Infusion of 125 µM ADPR, in contrast, induced an average current of 35 nA ± 6.4 nA in four out of four cells. Coinfusion of 8-Br-ADPR or 8-Br-cADPR both decreased the number of cells responding compared with the infusion of ADPR alone (due to the bimodal distribution, the fraction of cells responding with a current >1 nA was tested using Fisher’s exact test), indicating that both 8-Br-ADPR and 8-Br-cADPR antagonised activation of TRPM2 by ADPR or that 8-Br-cADPR is rapidly hydrolysed in the patched cells to yield the TRPM2 antagonist 8-Br-ADPR.

### 2.3. Activation of Human TRPM2 by ADPR and 2′-deoxy-ADPR Requires Both the MHR1/2 and the NUDT9H Domain

**Figure 3:**
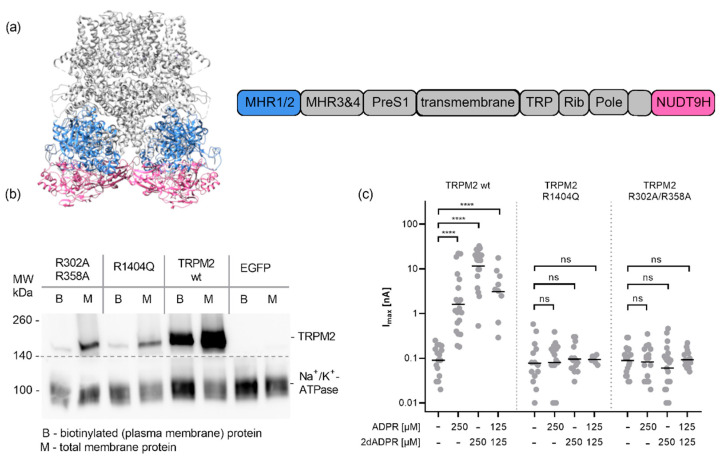
Effect of mutations in the MHR1/2 domain and in the NUDT9H domain on activation of TRPM2 by ADPR and 2′-deoxy-ADPR. (**a**) Structure of TRPM2, visualization of PDB:6PUS [18] generated in UCSF chimera [34], MHR1/2 (blue) and NUDT9H (magenta) domains are highlighted. (**b**) Effect of mutations on the plasma membrane localisation of TRPM2. Transiently transfected HEK293 cells (as above, additionally, empty pIRES-EGFP vector control was used) were treated with a nonmembrane permeant biotinylating agent. Cells were harvested and total membrane proteins isolated (M). The biotinylated proteins were enriched using neutravidin agarose beads. After separation on a 4–15% SDS-PAGE proteins were transferred to a PVDF membrane and detected by chemoluminescence using primary antibodies against human TRPM2 (upper part, NB 500-241, Novus Biologicals) and human Na^+^/K^+^-ATPase ((lower part, #3010, Cell Signalling Technology) and an HRP-conjugated secondary antibody, both parts, Dianova #111-035-045) (**c**) HEK293 cells were either transiently transfected with expression vectors for human TRPM2 with mutations in either the NUDT9H domain (R1404Q) or in the MHR1/2 domain (R30/R358A) or the wild type channel. 24 h post-transfection cells were placed in a bath solution with NMDG and infused via the patch pipette with an intracellular solution buffered to a Ca^2+^ of 200 nM with EGTA and containing either ADPR, 2′-deoxy-ADPR or a combination of both. Max currents at +15 mV obtained from repetitive voltage ramps are shown on a log scale. Log-transformed data were tested against the respective control by one-way ANOVA followed by a post hoc t test with Bonferroni correction; the mean is indicated by a horizontal bar (**** *p* < 0.0001, ns not significant).

The cryo-EM structure of TRPM2 from zebrafish (drTRPM2) led to the identification of a novel nucleotide-binding site in the N-terminus of the channel [17] that had previously been proposed by Kühn et al. based on their analysis of TRPM2 from *Nematostella vectensis* (nvTRPM2) [31]. In one of the cryo-EM structures from human TRPM2 (PDB: 6PUU), this site in the MHR1/2 was occupied by 8-Br-cADPR, which could indicate that this is also the binding site for cADPR [18] (especially when considering that 8-Br-cADPR has been described to specifically antagonize TRPM2 activation by cADPR but not by ADPR [9]). To investigate the role of the two domains (Figure 3a) for activation of human TRPM2 by nucleotides, we introduced point mutations in either site into our expression construct for human TRPM2 and transfected HEK293 cells with these constructs. Mutation of the MHR1/2 N-terminal residues R302/R358 to alanine (R278/R334 in drTRPM2) was shown to affect the binding of ADPR to the MHR1/2 domain, [17] whereas the C-terminal mutation R1404Q affects binding of ADPR to the NUDT9H domain of the channel [32]. We confirmed the expression of the mutated channels in the plasma membrane by surface biotinylation. While expression levels are somewhat lower for the mutants than for the wild type channel, both variants were found in the plasma membrane (Figure 3b). Whole-cell patch clamp experiments showed that the wildtype channel could be activated by either ADPR (4.7 nA ± 1.6 nA, *n* = 20) or the superagonist 2′-deoxy-ADPR, which resulted in a larger current (16.2 nA ± 2.1 nA, *n* = 20) (Figure 3c), as we have previously described [33]. When a combination of both agonists was infused, an intermediate current was observed (5.4 nA ± 1.8 nA, *n* = 9) (Figure 3c). The point mutations in either the MHR1/2 domain or the NUDT9H domain fully inactivated the channel, making it unresponsive to either agonist (Figure 3b), indicating that both domains are required for activation by nucleotide ligands.

### 2.4. The Isolated MHR1/2 Domain of TRPM2 Binds ADPR and 8-Br-ADPR, but Neither cADPR Nor 8-Br-cADPR

**Figure 4:**
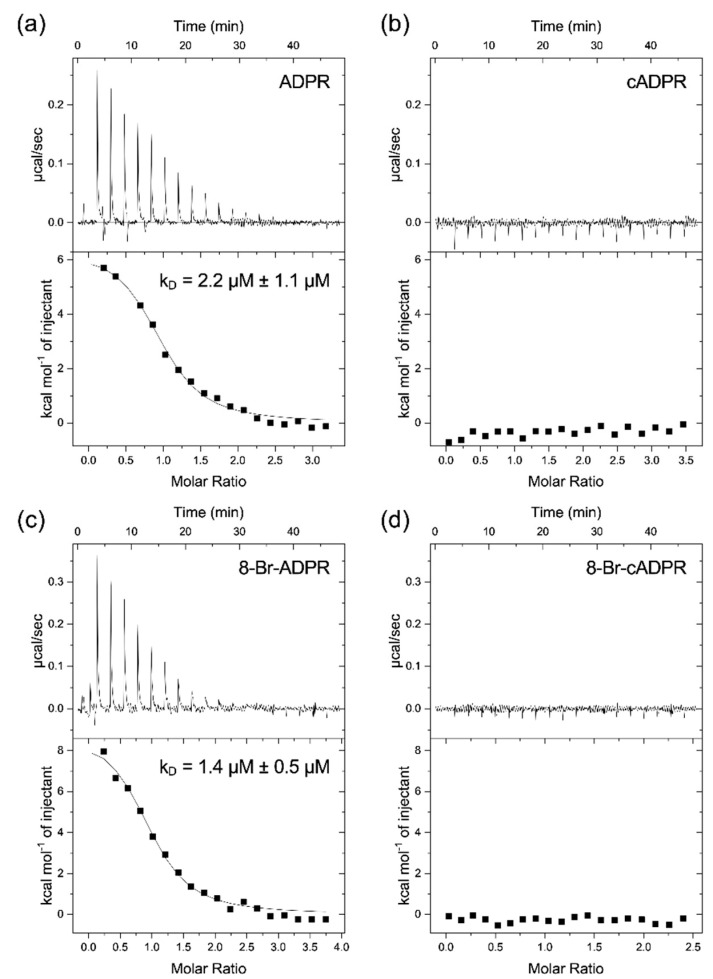
Binding of ADPR, cADPR, 8-Br-ADPR, and 8-Br-cADPR to the isolated MHR1/2 domain from drTRPM2. The isolated MHR1/2 domain from drTRPM2 was expressed in *E. coli* and purified by affinity and size exclusion chromatography and used to determine the binding affinity by isothermal titration calorimetry. The figure shows representative raw data and integrated peaks from 3 experiments per condition (**a**) ADPR, (**b**) cADPR, (**c**) 8-Br-ADPR, and (**d**) 8-Br-cADPR. The K_D_ value is expressed as mean ± SEM (*n* = 3). Raw data from individual experiments can be found in Supplementary Material Figure S2.

The observation that the MHR1/2 domain is required for activation of the channel by nucleotides and that no channel activation by cADPR was observed raises the question of whether the MHR1/2 domain is really capable of binding cADPR. To test this, we expressed the isolated MHR1/2 domain from drTRPM2 in *E. coli* and purified it by affinity chromatography and size exclusion chromatography. The isolated domain was then used to measure the binding of the nucleotides ADPR and cADPR by isothermal titration calorimetry (ITC). While we observed endothermal binding of ADPR to the isolated MHR1/2 domain with a K_D_ of 2.2 µM ± 1.2 µM (*n* = 3, Figure 4a), cADPR clearly did not bind to this domain under the same conditions (*n* = 3, Figure 4b). In addition, we measured the binding of the inhibitory analogues 8-Br-ADPR and 8-Br-cADPR. As we expected from the inhibitory activity, the linear nucleotide 8-Br-ADPR binds to the domain with a K_D_ similar to that of ADPR (1.4 µM ± 0.5 µM (*n* = 3, Figure 4c)). Interestingly, the cyclic 8-Br-cADPR did not bind under these conditions (*n* = 3, Figure 4d).

## 3. Discussion

Our interest in cADPR as a potential TRPM2 agonist was raised by a recent structural study that showed 8-Br-cADPR bound to the N-terminal MHR1/2 domain of TRPM2 (PDB 6PUU, [18]). In conjunction with the observation that ADPR probably folds into a horseshoe-like conformation when bound to this domain, and the older observation that 8-Br-cADPR selectively inhibits activation of TRPM2 by cADPR [9], together this may indicate that the MHR1/2 domain is a binding site for cADPR.

Using the purest cADPR preparation available to us, we tested cADPR for activation of human TRPM2 in whole-cell patch clamp experiments but observed no activation of TRPM2 regardless of temperature, Ca^2+^ buffering, or extracellular cation composition. We also observed no additive/synergistic effect of cADPR when coapplied with ADPR at a subthreshold concentration under conditions of low Ca^2+^ buffering. These findings are in agreement with the previous reports that hydrolysis of contaminating ADPR using a pyrophosphatase abrogates any effects of cADPR on TRPM2 [13,14]. Yu et al. recently proposed binding of cADPR to the ADPR-binding pocket in the C-terminal NUDT9H domain based on MD simulations [19]. To cope with the problem of ADPR contamination, they either synthesised cADPR themselves or purified cADPR by HPLC. Interestingly, the analysis by HPLC/ESI-MS included with the paper shows, besides the molecule ion peak for cADPR (*m*/*z* 540, the molecular weight of cADPR 541 Da,) also a smaller peak (*m*/*z* 558) which the authors attribute to a complex of cADPR + water (541 Da + 18 Da = 559 Da), but which in our opinion more likely is ADPR (m.w. 559 Da). The EC_50_ value for cADPR in this study was significantly larger than that for ADPR (250 µM for cADPR vs. 40 µM for ADPR), which likely is consistent with ADPR contamination of their cADPR preparation. Our patch clamp experiments under nearly identical buffer conditions (NaCl-based bath solution, intracellular buffer with low Ca^2+^ buffering) exhibited no activation of human TRPM2 by cADPR.

This posed the question of the role of the MHR1/2 domain for the activation of human TRPM2. While the MHR1/2 domain is clearly required for activation of TRPM2 from *Nematostella vectensis* and *Danio rerio*, there has been some dispute whether this also holds true for the human orthologue of TRPM2. While in their first cryo-EM structure of human TRPM2, Wang et al. did not detect ADPR in the MHR1/2 domain and therefore proposed that the human channel does not require binding of ADPR to this domain [35], Huang et al. later showed that ADPR binds to the MHR1/2 domain as well as the NUDT9H of human TRPM2. The observation that 8-Br-cADPR was only found in the N-terminal MHR1/2 domain indicates that there might be some differences in selectivity for the ligand and probably also a functional difference between the nucleotide-binding sites. As we proposed recently [36], this may mean that the sites are required for activation by different agonists. Since we observed no activation by cADPR, we tested variants of human TRPM2 with point mutations in the MHR1/2 and NUDT9H domain for activation by the agonist ADPR and the superagonist 2′-deoxy-ADPR. Our mutagenesis data show that activating human TRPM2 by both agonists requires both domains to be intact.

If both domains are required for activation by either agonist, this raises the question about the previous reports on the binding of 8-Br-cADPR to the MHR1/2 domain [18] and selective inhibition of cADPR-mediated activation of TRPM2 by 8-Br-cADPR [9]. Our HPLC analysis shows that, similar to cADPR, commercial preparations of 8-Br-cADPR can contain significant amounts of 8-Br-ADPR, a compound that we have previously shown to act as an ADPR antagonist. The local resolution of the cryo-EM structure at the MHR1/2 domain is not that high (PDB: 6PUU) and presumably does not allow to conclusively differentiate between an 8-Br-ADPR molecule in a horseshoe-like conformation and a cyclic 8-Br-cADPR molecule. Interestingly, our patch clamp data indicate that 8-Br-cADPR, similar to the linear analogue, does indeed inhibit activation of the channel by ADPR. However, while 8-Br-ADPR binds to the isolated MHR1/2 domain, 8-Br-cADPR exhibited no binding in our ITC experiments. This may indicate that 8-Br-cADPR is rapidly hydrolysed in the patched cells to the ADPR antagonist 8-Br-ADPR [29]. This leaves the question of whether cADPR does bind to the MHR1/2 domain, and our ITC data show conclusively that this is not the case.

In conclusion, while both nucleotide-binding sites, the N-terminal MHR1/2 domain as well as the C-terminal NUDT9H domain, are required for activation of human TRPM2 by the nucleotide agonists ADPR and 2′-deoxy-ADPR (2dADPR), we found no evidence for a role of the cyclic nucleotide cADPR in the activation of the human channel. The “cADPR antagonist” 8-Br-cADPR inhibits activation of the channel by ADPR, which might reflect hydrolysis to the ADPR antagonist 8-Br-ADPR, and the isolated MHR1/2 domain of TRPM2 binds with high-affinity binding to the TRPM2 agonist ADPR but does not bind to the cyclic nucleotide cADPR.

## 4. Materials and Methods

### 4.1. Cell Culture

Wildtype HEK293 cells were cultivated in DMEM (with 4.5 g/L glucose and without pyruvate, ThermoFisher Scientific, Waltham, MA, USA) with 10% fetal calf serum (Merck, Darmstadt, Germany), 100 units/mL penicillin and 100 µg/mL streptomycin (ThermoFisher Scientific, Waltham, MA, USA) For HEK 293 #24 (stably expressing TRPM2) 400 µg/mL G418 (Merck, Darmstadt, Germany) were added to the medium. The cells were kept at 37 °C and 5% CO_2_ and tested for mycoplasma contamination on a regular basis (using MycoAlert™ Mycoplasma Detection Kit, Lonza, Basel, Switzerland).

### 4.2. HPLC Analysis of Nucleotides

Nucleotides were checked by ion pair reverse-phase HPLC (RP-HPLC) as described before [33]. Samples of 1 nmol or 10 nmol of the nucleotides were analysed on a 1260 Infinity system (Agilent Technologies, Waldbronn, Germany) with a Multohyp BDS-C18 column (250 mm × 4.6 mm; particle size: 5 μm; CS Chromatographie Service, Langerwehe, Germany) protected by a guard cartridge (4 mm × 3 mm) containing a C18 ODS filter element (Phenomenex, Aschaffenburg, Germany). The mobile phase consisted of buffer A (20 mmol/L KH_2_PO_4_ and 5 mmol/L tetrabutylammonium dihydrogen phosphate, pH 6.0) with increasing amounts of methanol, resulting in the following gradient: 15% from start to 3 min, 31.25% at 11 min, 50% at 25 min, 50% at 27 min, 15% at 29 min, 15% at 38 min. The flow rate was 0.8 mL/min. Nucleotides were detected by their UV absorption at 260 nm and identified and quantified using external standards. Chromatograms were analysed offline using ChemStation Software (Revision C.01.05, Agilent Technologies, Waldbronn, Germany).

### 4.3. Site-Directed Mutagenesis

Construction of the expression vector for human TRPM2 (pIRES2-EGFP-hTRPM2) has been described before [15]. The mutation in the NUDT9H domain (R1404Q) was introduced using the QuikChange Site-Directed Mutagenesis Kit (Agilent Technologies, Waldbronn, Germany) using the primers 5′-CGGAAGCTGAAGCAGATCCTCCGGCAG-3′ and 5′-CTGCCGGAGGATCTGCTTCAGCTTCCG-3′. The coding sequence of pIRES2-EGFP-hTRPM2 R1404Q was confirmed by DNA sequencing (Eurofins Genomics, Ebersberg, Germany). The two mutations in the MHR1/2 (R302A/R358A) were introduced by a commercial service (Eurofins Genomics, Ebersberg, Germany).

### 4.4. Heterologous Expression and Electrophysiological Measurements (Patch Clamp)

Wild type HEK 293 cells were transfected with either pIRES-EGFP-TRPM2-wt (control), pIRES2-EGFP-hTRPM2-R302A/R358A, or pIRES2-EGFP-hTRPM2-R1404Q and 1 µg of vector DNA was incubated with 5 µL of JetPEI solution (polyPlus Transfection, Illkirch, France) in 150 mM NaCl solution (polyPlus Transfection, Illkirch, France) at a total volume of 250 µL for 30 min. The mix was added to Greiner 35 mm culture dishes (ThermoFisher Scientific, Waltham, MA, USA) containing 2.5 × 10^5^ cells in a cell culture medium as described above without antibiotics. Cells were kept at 37 °C and 5% CO_2_ overnight.

Before the electrophysiological experiments, the culture medium was removed, and cells were washed with the respective bath solution twice. During the experiments, the cells were kept in 1 mL of the bath solution. The standard bath solution was based on NMDG (140 mM N-methyl-D-glucamine (NMDG), 5 mM KCl, 5 mM D-Glucose, 10 mM HEPES, 3.3 mM MgCl_2_, 1 mM CaCl_2_, pH 7.4 at room temperature). For experiments at 37 °C, the temperature dependence of the buffer was taken into account and the pH adjusted to pH 7.63 at room temperature to make sure that the solution had a pH of 7.4 at 37 °C. In some experiments, the NMDG in the bath solution was replaced by 140 mM NaCl. 1.10 mm × 1.50 mm × 80 mm capillaries from borosilicate glass were pulled into pipettes with resistance between 1.5 and 3.5 MΩ using a P-97 horizontal puller (Sutter, Novato, CA, USA) and filled with pipette solution (120 mM KCl, 10 mM HEPES, 10 mM EGTA, 8 mM NaCl, 1 mM MgCl_2_, and 5.6 mM CaCl_2_, pH 7.2 at room temperature, resulting in a free Ca^2+^ concentration of 200 nM). For the experiments performed at 37 °C, the buffer contained 5.947 mM CaCl_2,_ and the pH was adjusted to 7.42 at room temperature to make sure the solution had 200 nM free Ca^2+^ and pH 7.2 at 37 °C. Ca^2+^ concentrations were calculated using MaxChelator. For some experiments, Ca^2+^ buffering was reduced. In these cases the pipette solution contained 129.3 mM KCl, 10 mM HEPES, 100 µM EGTA, 8 mM NaCl, and 1 mM MgCl_2_ at pH 7.2.

Whole-cell patch clamp experiments were performed using an EPC-10 amplifier (HEKA, Reutlingen, Germany) and PatchMaster software (v2x32, HEKA, Reutlingen, Germany). After the opening of the cells, the holding potential was set to −50 mV, and 90 voltage ramps between −85 and +20 mV (over 140 ms) were applied, one every 5 s. Compensation of series resistance was set to 70%. For the measurements that required temperature control, a peristaltic pump (miniplus2, Gilson, Limburg-Offheim, Germany) and a single channel heat controller (Warner Instruments, Holliston, MA, USA) were added to the setup, and the cells were continuously perfused with prewarmed bath solution at a flow rate of 0.5 mL/min. The temperature within the dish was checked using a thermocouple thermometer.

Adenine nucleotides (ADPR (SigmaAldrich, Taufkirchen, Germany), 2′-deoxy-ADPR (Biolog, Bremen, Germany), cADPR (Biolog, Bremen, Germany and SigmaAldrich, Taufkirchen, Germany), 8-Br-ADPR (Biolog, Bremen, Germany), 8-Br-cADPR (Biolog, Bremen, Germany and SigmaAldrich, Taufkirchen, Germany), and cIDPR (Biolog, Bremen, Germany)) were added to the pipette solution at the indicated concentrations.

### 4.5. Surface Biotinylation of Proteins of the Plasma Membrane

For each condition, one T25 flask of wild type HEK293 cells was prepared at 75% confluency and transfected using the Lipofectamine LTX and plus reagents (ThermoFisher Scientific, Waltham, MA, USA) using the TRPM2 expression vectors described in Section 4.3.

48 h post-transfection, successful transfection was confirmed by checking EGFP fluorescence by fluorescence microscopy. Then cells were washed three times with D-PBS (with Ca^2+^ and Mg^2+^) and treated with 1 mg/mL EZ-Link Sulfo-NHS-LC-Biotin (ThermoFisher Scientific, Waltham, MA, USA) for 30 min. Afterwards, cells were removed from the flask using 2 mM EGTA in PBS and collected by centrifugation (500× *g*). The cells were washed two times with PBS/EGTA, and membrane proteins were extracted using the ProteoExtract Native Membrane Protein Extraction Kit (Merck, Darmstadt, Germany). Total membrane protein samples were collected and stored at −20 °C; membrane extraction samples were mixed with NeutrAvidin Agarose beads (ThermoFisher Scientific, Waltham, MA, USA) overnight, shaking with 20 rpm at 4 °C, centrifugated (2500× *g*, 2 min at RT) and stored at −20 °C as well.

Both sample groups were mixed with 10 µL SDS sample buffer per sample, heated to 75 °C for 7 min, and centrifugated (500× *g*, 1 min at RT) and separated using a precast 4–15 % SDS gel at 200 V for ca. 40 min until the coloured band left the gel. The gel was transferred to a PVDF-Membrane Immobilon-P via Western blot using 25 mM Tris, 192 mM glycine, 20% methanol, 0.025% SDS transfer buffer. The membrane was cut at the 140 kDa marker band (Spectra Multicolor Broad Range Ladder, ThermoFisher Scientific, Waltham, MA, USA). Both parts were washed three times and blocked overnight using 5% milk powder in TBS-T at 4 °C. The upper membrane part was incubated with rabbit a-TRPM2-antibodies (NB 500-241, Novus Biologicals, Wiesbaden Nordenstadt, Germany) 1:50,000 in TBS-T containing 2.5% milk powder for 1 h at RT. Rabbit α-Na^+^/K^+^-ATPase-antibodies (#3010, Cell Signalling Technology, Frankfurt am Main, Germany) 1:10,000 were used on the lower membrane part at the same conditions. Membranes were washed in TBS-T three times for 5 min and incubated with HRP-conjugated goat anti-rabbit antibodies (#111-035-045, Dianova, Hamburg, Germany) 1:10,000 at the same conditions as before. Membranes were washed three times for 5 min in TBS-T.

SuperSignal West Dura chemiluminescence substrate (ThermoFisher Scientific, Waltham, MA, USA) was mixed 1:10 with SuperSignal West Pico chemiluminescence substrate (ThermoFisher Scientific, Waltham, MA, USA), put on the membranes for 5 min and carefully dried. The membrane was photographed at one picture per minute.

### 4.6. Expression and Purification of the Isolated MHR1/2 Domain from drTRPM2

A pnEK-vH vector was used to express the zebrafish TRPM2 MHR1/2 domain (drMHR1/2, residues 1-419 of drTRPM2) with an N-terminal His_6_-tag. *E. coli* BL21 Gold (DE3) cells were grown in Terrific Broth medium supplemented with 25 µg/mL kanamycin at 37 °C until a density of 0.8 (measured at 600 nm) was reached. Protein expression was induced by 0.1 mM isopropyl β-D-1-thiogalactopyranoside (IPTG) and carried out for 16 h at 20 °C. The cells were harvested by centrifugation, and lysis was carried out using a high-pressure homogenizer (EmulsiFlex-C3, Avestin, Ottawa, Ontario, Canada). The His_6_-tagged protein was purified from the clarified lysate using immobilised metal affinity chromatography (IMAC) and subsequent size exclusion chromatography on a Superdex S200 increase 10/300 column (Cytiva, Freiburg, Germany) using HEPES buffer (20 mM HEPES pH 7.5, 150 mM NaCl).

### 4.7. Isothermal Titration Calorimetry

Binding assays by isothermal titration calorimetry (ITC) were carried out at 25 °C on a MicroCal ITC-200 isothermal titration calorimeter (Malvern Panalytical, Malvern, UK). Thermodynamic parameters were analysed with the MicroCal ORIGIN^TM^ software (Additive GmbH, Friedrichsdorf, Germany). Adenine nucleotides were dissolved in HEPES buffer to a concentration of 300 µM and placed in the ITC syringe. Nineteen injections of the ligand (2 µL each) were titrated into 20 µM drMHR1/2 in the ITC sample cell. Each individual injection was interspaced by 150 s, and the stirring speed was set to 750 rpm. The ligand was titrated into HEPES buffer to allow for baseline correction. All measurements were performed as triplicates, and binding curves were fitted with a one-site binding model.

### 4.8. Statistical Analysis

Statistical analysis of the data was performed using GraphPad Prism (v8.0.1, GraphPad Software, San Diego, CA, USA). Data in the text are represented as mean ± SEM if not indicated otherwise. Since the currents obtained from whole-cell recordings were significantly skewed towards positive values, they were log-transformed to achieve normality. Normality of transformed data was confirmed by Kolmogorov–Smirnov normality test. Normal log-transformed data were tested by one-way ANOVA, using Bonferroni correction to adjust *p* values for multiple testing. The data acquired with decreased Ca^2+^ buffering in the pipette solution showed a bimodal distribution; since both modes showed the skewed distribution, these data were also log-transformed but tested using a nonparametric Kruskal–Wallis test with Dunn’s correction. In the case of the experiments with 8-Br-ADPR/8-Br-cADPR, a threshold was set between the modes, and the distributions were tested pairwise using Fisher’s exact test. In all cases, test results with *p* < 0.05 were considered to be significant.

## Data Availability

Data are available upon reasonable request from the corresponding author.

## Supplementary Materials

The following supporting information can be downloaded at: https://www.mdpi.com/article/10.3390/ijms23063163/s1.

## References

[1] Clapper D.L., Walseth T.F., Dargie P.J., Lee H.C. (1987). Pyridine nucleotide metabolites stimulate calcium release from sea urchin egg microsomes desensitized to inositol trisphosphate. J. Biol. Chem..

[2] Lee H.C. (2001). Physiological Functions of Cyclic ADP-Ribose and NAADP as Calcium Messengers. Annu. Rev. Pharmacol. Toxicol..

[3] Lee H.C. (2012). The Cyclic ADP-Ribose/NAADP/CD38-Signaling Pathway: Past and Present. Messenger.

[4] Galione A., Lee H.C., Busa W.B. (1991). Ca(2+)-induced Ca^2+^ release in sea urchin egg homogenates: Modulation by cyclic ADP-ribose. Science.

[5] Pérez C.F., Marengo J.J., Bull R., Hidalgo C. (1998). Cyclic ADP-ribose activates caffeine-sensitive calcium channels from sea urchin egg microsomes. Am. J. Physiol..

[6] Guse A.H. (2005). Second messenger function and the structure-activity relationship of cyclic adenosine diphosphoribose (cADPR). FEBS J..

[7] Guse A.H., Berg I., Da Silva C.P., Potter B.V.L., Mayr G.W. (1997). Ca^2+^ entry induced by cyclic ADP-ribose in intact T-lymphocytes. J. Biol. Chem..

[8] Thompson M., White T., Chini E.N. (2006). Modulation of store-operated Ca^2+^ entry by cyclic-ADP-ribose. Braz. J. Med. Biol. Res. (Rev. Bras. Pesqui. Med. Biol.).

[9] Kolisek M., Beck A., Fleig A., Penner R. (2005). Cyclic ADP-ribose and hydrogen peroxide synergize with ADP-ribose in the activation of TRPM2 channels. Mol. Cell.

[10] Perraud A.L., Fleig A., Dunn C.A., Bagley L.A., Launay P., Schmitz C., Stokes A.J., Zhu Q., Bessman M.J., Penner R. (2001). ADP-ribose gating of the calcium-permeable LTRPC2 channel revealed by Nudix motif homology. Nature.

[11] Guse A.H., da Silva C.P., Berg I., Skapenko A.L., Weber K., Heyer P., Hohenegger M., Ashamu G.A., Schulze-Koops H., Potter B.V. (1999). Regulation of calcium signalling in T lymphocytes by the second messenger cyclic ADP-ribose. Nature.

[12] Togashi K., Hara Y., Tominaga T., Higashi T., Konishi Y., Mori Y., Tominaga M. (2006). TRPM2 activation by cyclic ADP-ribose at body temperature is involved in insulin secretion. EMBO J..

[13] Heiner I., Eisfeld J., Warnstedt M., Radukina N., Jüngling E., Lückhoff A. (2006). Endogenous ADP-ribose enables calcium-regulated cation currents through TRPM2 channels in neutrophil granulocytes. Biochem. J..

[14] Tóth B., Csanády L. (2010). Identification of direct and indirect effectors of the transient receptor potential melastatin 2 (TRPM2) cation channel. J. Biol. Chem..

[15] Fliegert R., Riekehr W.M., Guse A.H. (2020). Does Cyclic ADP-Ribose (cADPR) Activate the Non-selective Cation Channel TRPM2?. Front. Immunol..

[16] Kühn F.J.P., Kühn C., Lückhoff A. (2015). Functional characterisation of a TRPM2 orthologue from the sea anemone Nematostella vectensis in human cells. Sci. Rep..

[17] Huang Y., Winkler P.A., Sun W., Lü W., Du J. (2018). Architecture of the TRPM2 channel and its activation mechanism by ADP-ribose and calcium. Nature.

[18] Huang Y., Roth B., Lü W., Du J. (2019). Ligand recognition and gating mechanism through three ligand-binding sites of human TRPM2 channel. Elife.

[19] Yu P., Liu Z., Yu X., Ye P., Liu H., Xue X., Yang L., Li Z., Wu Y., Fang C. (2019). Direct Gating of the TRPM2 Channel by cADPR via Specific Interactions with the ADPR Binding Pocket. Cell Rep..

[20] Moreau C., Kirchberger T., Swarbrick J.M., Bartlett S.J., Fliegert R., Yorgan T., Bauche A., Harneit A., Guse A.H., Potter B.V.L. (2013). Structure-activity relationship of adenosine 5′-diphosphoribose at the transient receptor potential melastatin 2 (TRPM2) channel: Rational design of antagonists. J. Med. Chem..

[21] McHugh D., Flemming R., Xu S.-Z., Perraud A.-L., Beech D.J. (2003). Critical intracellular Ca^2+^ dependence of transient receptor potential melastatin 2 (TRPM2) cation channel activation. J. Biol. Chem..

[22] Starkus J., Beck A., Fleig A., Penner R. (2007). Regulation of TRPM2 by extra- and intracellular calcium. J. Gen. Physiol..

[23] Csanády L., Törocsik B. (2009). Four Ca^2+^ ions activate TRPM2 channels by binding in deep crevices near the pore but intracellularly of the gate. J. Gen. Physiol..

[24] Wagner G.K., Black S., Guse A.H., Potter B.V.L. (2003). First enzymatic synthesis of an N1-cyclised cADPR (cyclic-ADP ribose) analogue with a hypoxanthine partial structure: Discovery of a membrane permeant cADPR agonist. Chem. Commun..

[25] Wagner G.K., Guse A.H., Potter B.V.L. (2005). Rapid synthetic route toward structurally modified derivatives of cyclic adenosine 5′-diphosphate ribose. J. Org. Chem..

[26] Kirchberger T., Wagner G., Xu J., Cordiglieri C., Wang P., Gasser A., Fliegert R., Bruhn S., Flügel A., Lund F.E. (2006). Cellular effects and metabolic stability of N1-cyclic inosine diphosphoribose and its derivatives. Br. J. Pharmacol..

[27] Walseth T.F., Lee H.C. (1993). Synthesis and characterization of antagonists of cyclic-ADP-ribose-induced Ca^2+^ release. Biochim. Biophys. Acta.

[28] Sethi J.K., Empson R.M., Bailey V.C., Potter B.V.L., Galione A. (1997). 7-deaza-8-bromo-cyclic ADP-ribose, the first membrane-permeant, hydrolysis-resistant cyclic ADP-ribose antagonist. J. Biol. Chem..

[29] Partida-Sanchez S., Gasser A., Fliegert R., Siebrands C.C., Dammermann W., Shi G., Mousseau B.J., Sumoza-Toledo A., Bhagat H., Walseth T.F. (2007). Chemotaxis of mouse bone marrow neutrophils and dendritic cells is controlled by adp-ribose, the major product generated by the CD38 enzyme reaction. J. Immunol..

[30] Tóth B., Iordanov I., Csanády L. (2020). Selective profiling of N- And C-terminal nucleotide-binding sites in a TRPM2 channel. J. Gen. Physiol..

[31] Kühn F.J.P., Kühn C., Winking M., Hoffmann D.C., Lückhoff A. (2016). ADP-Ribose Activates the TRPM2 Channel from the Sea Anemone Nematostella vectensis Independently of the NUDT9H Domain. PLoS ONE.

[32] Perraud A.-L., Takanishi C.L., Shen B., Kang S., Smith M.K., Schmitz C., Knowles H.M., Ferraris D., Li W., Zhang J. (2005). Accumulation of free ADP-ribose from mitochondria mediates oxidative stress-induced gating of TRPM2 cation channels. J. Biol. Chem..

[33] Fliegert R., Bauche A., Wolf Pérez A.-M., Watt J.M., Rozewitz M.D., Winzer R., Janus M., Gu F., Rosche A., Harneit A. (2017). 2′-Deoxyadenosine 5′-diphosphoribose is an endogenous TRPM2 superagonist. Nat. Chem. Biol..

[34] Pettersen E.F., Goddard T.D., Huang C.C., Couch G.S., Greenblatt D.M., Meng E.C., Ferrin T.E. (2004). UCSF Chimera—A visualization system for exploratory research and analysis. J. Comput. Chem..

[35] Wang L., Fu T.-M., Zhou Y., Xia S., Greka A., Wu H. (2018). Structures and gating mechanism of human TRPM2. Science.

[36] Fliegert R., Hölzer H.T., Guse A.H. (2018). TRPM2 activation: Paradigm shifted?. Cell Calcium.

