# Supplementary material for: cADPR Does Not Activate TRPM2
Source: Int J Mol Sci. 2022 Mar 15;23(6):3163. doi: 10.3390/ijms23063163 (PMC8949931; doi:10.3390/ijms23063163)
Supplement: Supplementary file 1 [file ijms-23-03163-s001.zip › ijms-1581652-supplementary.pdf]

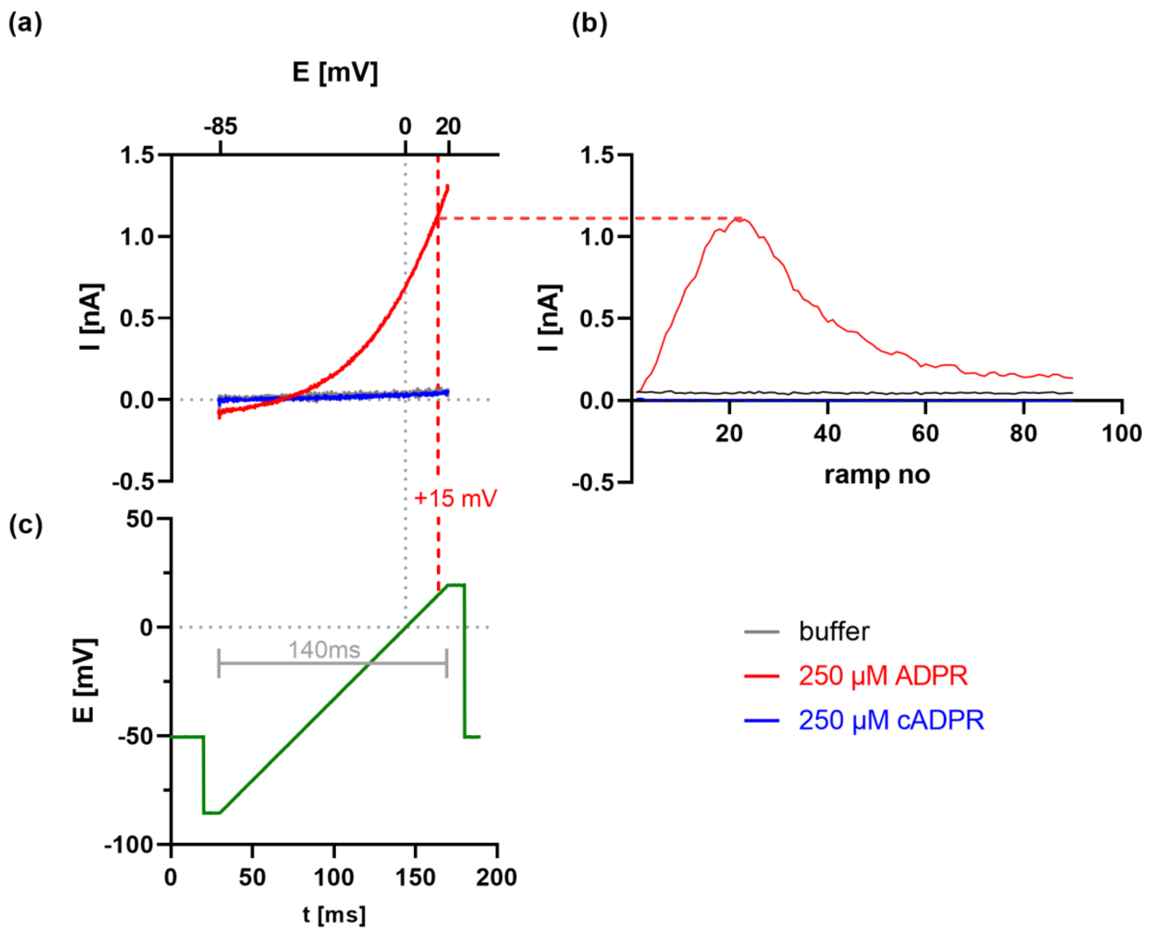

Supplementary Figure S1: Exemplary whole cell patch clamp experiments (a and b) Whole cell patch clamp experiments were performed with the patch pipette containing buffer alone, or buffer containing 250 µM ADPR or 125 µM cADPR. A total of 90 voltage ramps from -85 mV and +20 mV (over 140ms) were applied every 5 seconds. (a) The current-volt-relationship obtained from representative ramps displaying maximal current at +15 mV within their measurement. (b) Time course of the maximum current at +15 mV from representative recordings. (c) Schematic representation of the voltage ramps applied during the recordings.

(a)

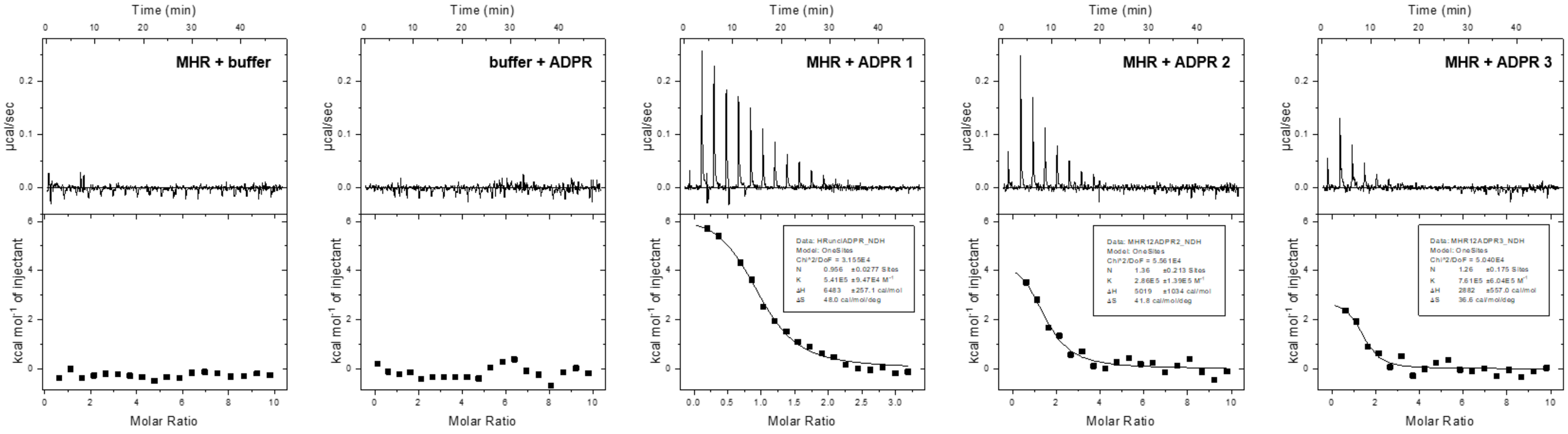

MHR + buffer
buffer + ADPR
MHR + ADPR 1
MHR + ADPR 2
MHR + ADPR 3
Time (min)
µcal/sec
kcal mol-1 of injectant
Molar Ratio
Data: HRunclADPR_NDH
Model: OneSites
Chi^2/DoF = 3.155E4
N 0.956 ±0.0277 Sites
K 5.41E5 ±9.47E4 M-1
ΔH 6483 ±257.1 cal/mol
ΔS 48.0 cal/mol/deg
Data: MHR12ADPR2_NDH
Model: OneSites
Chi^2/DoF = 5.561E4
N 1.36 ±0.213 Sites
K 2.86E5 ±1.39E5 M-1
ΔH 5019 ±1034 cal/mol
ΔS 41.8 cal/mol/deg
Data: MHR12ADPR3_NDH
Model: OneSites
Chi^2/DoF = 5.040E4
N 1.26 ±0.175 Sites
K 7.61E5 ±6.04E5 M-1
ΔH 2882 ±557.0 cal/mol
ΔS 36.6 cal/mol/deg

(b)

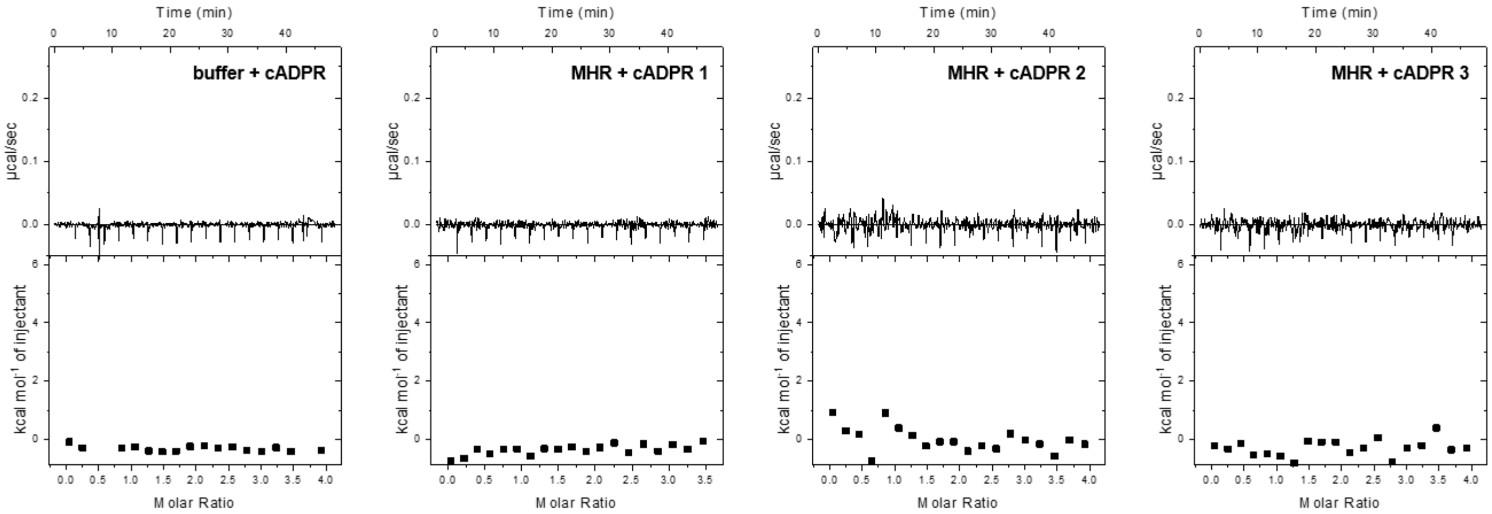

buffer + cADPR
MHR + cADPR 1
MHR + cADPR 2
MHR + cADPR 3
Time (min)
µcal/sec
kcal mol-1 of injectant
Molar Ratio

(c)

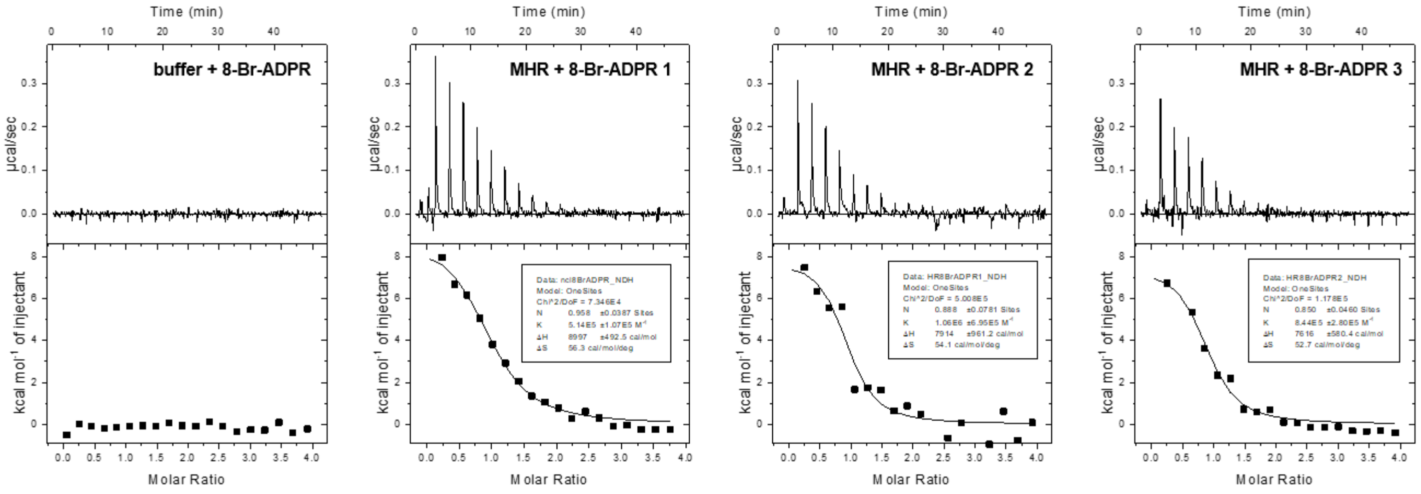

(d)

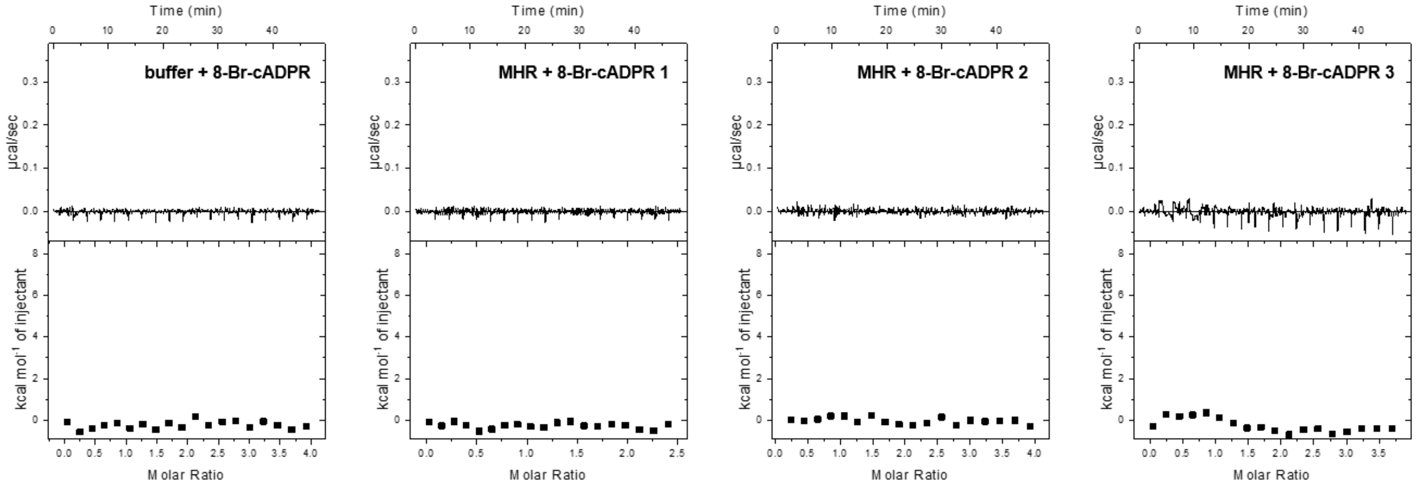

Supplementary Figure S2: Individual ITC Experiments. Binding of (a) ADPR, (b) cADPR, (c) 8-Br-ADPR and (d) 8-Br-cADPR to the isolated MHR1/2 domain of drTRPM2 was determined by isothermal titration calorimetry. Negative controls by titration of buffer to the MHR1/2 domain as well as titration of the ADPR analogues to the buffer are included.
